# “Feeling Safe, Feeling Seen, Feeling Free”: Combating stigma and creating culturally safe care for sex workers in Chicago

**DOI:** 10.1371/journal.pone.0253749

**Published:** 2021-06-29

**Authors:** Randi Beth Singer, Amy K. Johnson, Natasha Crooks, Douglas Bruce, Linda Wesp, Alexa Karczmar, Lucy Mkandawire-Valhmu, Susan Sherman

**Affiliations:** 1 Department of Human Development Nursing Science, College of Nursing University of Illinois at Chicago, Chicago, Illinois, United States of America; 2 Division of Adolescent and Young Adult Medicine, Ann & Robert H. Lurie Children’s Hospital of Chicago, Chicago, Illinois, United States of America; 3 Department of Pediatrics, Northwestern University Feinberg School of Medicine, Chicago, Illinois, United States of America; 4 Department of Health Sciences, College of Science and Health, DePaul University, Chicago, Illinois, United States of America; 5 College of Nursing, University of Wisconsin-Milwaukee, Milwaukee, Wisconsin, United States of America; 6 Department of Health, Behavior and Society, John Hopkins Bloomberg School of Public Health, Baltimore, Maryland, United States of America; University of Perugia, ITALY

## Abstract

**Background:**

Previous studies have established that sex workers experience discrimination and stigma within healthcare settings, limiting their access and receipt of culturally safe care. These barriers impact sex workers’ ability and desire to routinely engage with the healthcare system. Community empowerment interventions that are culturally safe offer an effective strategy to improve access to services and health outcomes for sex workers.

**Objectives:**

This project was designed to inform the development of community empowerment interventions for sex workers by understanding their self-management, health promotion, and harm reduction needs.

**Methods:**

In-depth interviews (N = 21) were conducted with sex workers in Chicago. Transcripts of individual interviews were analyzed in Dedoose using rapid content analysis.

**Results:**

Participants had a mean age of 32.7 years; 45% identified as White, 20% as Black, 15% as Latinx, and 20% as multiple races; 80% identified as Queer. A total of 52% of participants identified as cisgender women, 33% as transgender or gender fluid, 10% as cisgender men, and 5% declined to answer. Themes of self-management practices, stigmatizing and culturally unsafe experiences with healthcare providers, and the prohibitive cost of healthcare emerged as consistent barriers to routinely accessing healthcare. Despite identifying patient-centered care as a desired healthcare model, many participants did not report receiving care that was respectful or culturally responsive. Themes also included developing strategies to identify sex worker-safe care providers, creating false self-narratives and health histories in order to safely access care, and creating self-care routines that serve as alternatives to primary care.

**Conclusion:**

Our findings demonstrate how patient-centered care for sex-workers in Chicago might include holistic wellness exercises, accessible pay scales for services, and destigmatizing healthcare praxis. Focus on culturally safe healthcare provision presents needs beyond individualized, or even community-level, interventions. Ongoing provider training and inbuilt, systemic responsivity to patient needs and contexts is crucial to patient-centered care.

## Introduction

According to the World Health Organization, sex workers include adults of all gender identities who exchange money, resources, or survival needs for sexual services [1]. Complex structural and social factors (i.e., stigma, discrimination and criminalization) create unique challenges to targeting health promotion efforts for sex workers and contribute to health inequities such as HIV [2]. Sex workers may regularly experience the social repercussions of such stigmatized work. Fear of discrimination within healthcare settings, violence, stigma or harassment limits access to prevention information, services, and treatment [3–7]. Criminalization, targeted policing, environments that restrict worker agency, and poverty further impact sex workers’ abilities to practice harm reduction and health promotion behaviors and limit patient ability to disclose sex work [3,4,8,9]. Health inequities and barriers to care experienced by sex workers are a social justice issue with systemic level foundations, challenges, and solutions [10]. This paper seeks to understand the range of experiences of care by those engaged in sex work to inform culturally safe care.

Sex workers experience barriers in accessing culturally safe health services at policy, community, institutional, interpersonal, and intrapersonal levels [10]. Community-level contexts such as stigma experienced in sex work and interpersonal contexts such as fear of discrimination contributes to such obstacles to care for sex workers. However, global research has shown that community empowerment models align with patient-centered care and concretely address the structural and social barriers to care among sex workers [11–13]. Community empowerment models acknowledge that health inequities are fundamentally structural, and that efforts to improve the health and well-being of those most marginalized require addressing structural violence at multiple levels [11,12,14]. Interventions developed through community empowerment are designed, implemented, and assessed by sex workers and are based on their experiences, knowledge, and collective leadership [2,11,12,15]. The aim of such interventions is to decrease biologic vulnerability to HIV, as well as social and structural barriers while increasing individual, financial, and community resources and social support [11,16]. Importantly, these approaches view sex workers as part of the solution and seek to holistically support them in achieving their own health and human rights, such as access to safe and reliable patient-centered care [1,17].

With decreased disclosure, patients are not offered the prevention information, services, and treatment that aligns with their health needs [5,18]. Although healthcare providers may be experts in diagnosis, treatment and prevention, patients are the experts in their own lives and bring with them a wealth of lived experience. A respectful partnership between care provider and patient serves to directly address patient needs and reduce barriers to care [18]. Research demonstrates both physical and mental health benefits of patient-centered care [19,20].

Meaningful patient-centered care requires that providers engage in culturally safe practices which transfers power from the provider to the patient through partnership [19]. “Cultural Safety involves understanding history, safety needs, power imbalances, the influence of staff values and beliefs on service delivery” [21]. The provider who practices culturally safe care seeks to understand the patients’ lived experiences and incorporate them into clinical care [18,22,23]. Understanding clinical care through a cultural safety framework will provide a comprehensive approach to the management of the whole patient in line with patient-centered care because it is the patient who determines what is safe [18]. This project aims to utilize community empowered research to facilitate culturally safe care for sex workers.

## Methods

The study took place from April 2020 through June 2020. Participants were passively recruited through clinic-based flyers, social media (i.e., Twitter, Facebook, Instagram), private community list-servs and actively recruited via word-of-mouth referrals. Potential participants emailed or called the study team to assess eligibility and learn more about the study; if eligible and interested a remote study visit was scheduled. To be eligible to participate in the study, participants had to be: a) age 18 or older; b) exchanged oral, vaginal, or anal sex for something of value in the past 12 months; c) live in the Chicago area; d) speak and understand English; and e) be willing and able to provide informed consent. The institutional review boards of University of Illinois Chicago and Howard Brown Health approved all study procedures.

### Data collection

Individual interviews lasted between 45–90 minutes and were conducted using a secure institutional instance of Zoom video conference platform. Current and former sex workers (peers) were trained as qualitative interviewers and used a semi-structured interview guide that was developed in collaboration with community stakeholders. [24,25]. The semi-structured interview guide was informed by the literature and previous research, and covered physical, sexual and emotional health, experiences with healthcare, HIV/STI prevention, and harm reduction techniques [26–29]. Additionally, the guide contained questions and example probes but allowed interviewers to add probes as needed and to explore topics naturally as they emerged. For example, we asked “Can you tell me about what it’s like when you go to see healthcare providers?” to elicit responses about their healthcare experience. In-group knowledge enriched interviewers’ ability to meaningfully engage in conversation about healthcare barriers during the qualitative interviews when we asked questions such as, “What is it was like to disclose participation in sex work to healthcare providers?”. We ended the interview by asking all participants the following questions: “How can health providers and doctor’s offices do better for you? What health issues are most important to you and why?” We included these specific questions in order to tailor future interventions to suit the stated needs of the community. In addition to post-session debriefings, and review of transcribed audio of the interviews with peer interviewers, the first author (RBS) attended a subset of interviews to ensure fidelity to the interview guide and add probes as necessary [25].

### Data analysis

The coding team was comprised of two qualitative researchers (AK, AKJ) with substantial experience in both methods and content area. A codebook was developed through an iterative process using the interview guide and a first review of interview transcripts. The primary coder applied initial codes to a subset of transcripts; both coders reviewed these transcripts and revised codes to reflect patterns in the data. The codebook was continuously updated with definitions and example quotes. Dedoose, a flexible online mixed-methods platform, was used for qualitative analysis [30]. Inter-rater reliability (IRR) was established using the “test” feature within Dedoose, which randomly selects a subset of transcripts and allows for blinded double coding [31]. The coding team achieved an IRR of at least 0.80 across all codes. Rapid content analysis was employed to identify themes and sub-themes in the dataset [32]. The coding team presented preliminary findings and discussed the inclusion of both major and minor (diverse and/or outlier) themes apparent in the data with the entire study team made up of current and former sex workers, healthcare providers, and research faculty to ensure validity and relevance. Patterns in themes, including consistent repetition and limited new topics, ensured saturation was achieved.

## Results

### Participant characteristics

Participants (N = 21) had a mean age of 32.7 years and ranged in age from 20 to 46 years. Participants were racially/ethnically diverse as 45% identified as White, 20% as Black, 15% as Latinx, and 20% as multiple races (Table 1). In terms of gender identity, 55% identified as cisgender women, 20% as gender queer, 15% as transgender women, and 10% as cisgender men. The majority of the sample reported sexual minority identities with 81% identifying as queer, bisexual, pansexual, or gay. The sample was highly educated with 80% reporting college degrees or higher education. Fifty-seven percent of respondents described sex work as their main source of income. All participants reported a visit to a health care provider within the last 24 months, with the majority within the past 12 months. Thirty-three percent of participants reported having no health insurance.

**Table 1:** Demographics of interviewed Chicago sex workers (N = 21).

**Age**	Mean Age	32.7 years old
**Race/ethnicity**	White (N = 10)	48%
	Black (N = 4)	19%
	Latinx (N = 3)	14%
	Asian (N = 1)	5%
	Multiple Races (N = 3)	14%
**Gender identity**	Cisgender Woman (N = 11)	52%
	Gender Queer (N = 4)	19%
	Transgender Woman (N = 3)	14%
	Cisgender Man (N = 2)	10%
	Prefer not to disclose (N = 1)	5%
**Sexual identity**	Queer, Bisexual, Pansexual, Gay (N = 17)	81%
	Heterosexual (N = 3)	14%
	Asexual (N = 1)	5%
**Education**	College /Advanced Degree (N = 13)	62%
	Some College (N = 4)	19%
	High School Degree (N = 3)	14%
	GED (N = 1)	5%

Study participants reported limited experiences with culturally safe patient-centered healthcare, with the majority of participants characterizing their experiences as stigmatizing and judgmental. These participants described uncomfortable and harmful interactions with healthcare providers. Further, many participants reported not disclosing their identity as a sex worker to healthcare providers to prevent stigma and judgement. Others described opting to use holistic wellness (e.g., yoga, meditation) to maintain health rather than institutionalized and routine healthcare. Several themes were identified while discussing healthcare experiences, including stigmatization and cultural safety, condescension, disregard and distrust, and financial barriers to accessing needed services. We have provided a frequency table (S1 Table) demonstrating how many of the 21 total participants reported experiencing each theme.

#### Stigmatization & cultural safety

The majority of participants (n = 16) reported experiences of stigmatization when accessing healthcare. As a result, some chose not to share their background in sex work, limiting their ability to meaningfully engage with providers and receive comprehensive care. Some were explicitly “discouraged from returning” for future care (multi-racial transwoman, 34 years old). Others noticed an immediate change in the demeanor and attitudes of their providers after they disclosed their employment as a sex worker. One participant described such an experience:

“*I’ve had a couple negative experiences there with nurses*, *which surprised me… You know*, *they want to know what medications you’re on*, *and you start to tell them*, *and you can see their faces … if you’re on a mood stabilizer*, *and then as soon as you say Truvada*, *they’ve written you off as totally useless and you know disease ridden*.”

(ID 14, white ciswoman, 39 years old)

Other participants described sharing partial truths or giving providers limited information, as a way to mitigate stigma and judgement from healthcare providers:

“*I think usually like when I’m seeing like a primary care physician or something*. *I’ll kind of test the waters*. *Or I’ll give them*, *you know*, *half of it […] Because usually*, *I feel like it’s just I think that we face so much stigma and you would want to hope and be in good hopes that*, *you know*, *a health care provider would treat you without bias […] so sometimes I feel like it’s I’m better off by just not disclosing it*.”

(ID 02, multiracial ciswoman, 26 year old)

This stigmatization made participants hesitant to share their experiences engaging in sex work as well as seeking out health care services. Many discussed this stigmatization as the reason for limiting open communication with care providers and pointed to experiences of stigma as a significant deterrent from accessing health care resources. This cycle of stigmatization and corresponding decreased disclosure sheds light on the underlying systemic issue preventing culturally safe care for sex workers. Another participant described not feeling comfortable disclosing their work due to past experiences of stigma related to their intersecting identities.

“*I do not disclose that I’m a worker*, *because I fit into enough subcategories of society to already be stigmatized by the healthcare industry*. *So I don’t need another thing on top of that*, *and it’s not their business*.”

(ID 1, Black ciswoman, 28 year old)

#### Dismissal, disregard & distrust

Participants reported being condescended to, disbelieved and disregarded by medical providers. In some cases, this distrust in sex workers’ understanding of their own experiences harmed patient health. One participant shared:

“*Yeah*. *I’ve been dismissed a lot*. *One time I went in and told them I had a UTI and they were like*, *‘No*, *you don’t*.*’ I was like*, *‘Yeah*, *I do*.*’ And then I went home and then I had to go to urgent care later*, *because I was peeing blood*.”

(IDI 2, white ciswoman, 30 year old,)

In cases where these concrete examples of provider distrust were not given, participants reported a general experience of distrust, disregard and condescension. When asked what providers could do better for the community, one participant shared:

“.. *believe people[…] do not make assumptions about people just in general […] Treat everybody decently across the board and believe them*, *if maybe take a second…if someone says that they’re experiencing something and you have the urge to think that they’re exaggerating*, *take a second to ask yourself why you think that they are exaggerating and if they looked a little different*, *or if they were different gender or if they were a little older or younger*, *would you assume*, *would you also deny them what they’re requesting*?”

(IDI 1, Black ciswoman, 28 year old)

Participants lack of lack of trust in providers resulted in many of them choosing not to engage in healthcare or questioning providers expertise/decisions in critical moments. In weighing the decision of whether to go to a medical provider to address health issues, one participant noted:

“*I think that we face so much stigma and you would want to hope … that*, *you know*, *a healthcare provider would treat you without bias*, *but that’s also a risk that you don’t really want to take*. *And so sometimes I’m just like*, *will it cost me more trouble*?”

(IDI 02, multiracial ciswoman, 26 year old)

Distrust was also fueled by experiences of racism at the interpersonal and institutional level. Participants of non-white identity spoke to this point by acknowledging their hesitancy to access care from a system grounded in racial oppression and discrimination. Many discussed how the healthcare system has not only failed to protect Black and Brown bodies, but they pointed to Tuskegee Syphilis Experiment and Henrietta Lacks as tangible reasons for limiting engagement with care providers. Additionally, many participants spoke about the bias in care they experienced based on their identities. Identifying as transgender also aligned with participant distrust of care providers. Transgender participants suggested that in the past, care providers focused more on their transgender identity or sex worker identity than on the reason for their visit. For this reason, they were less inclined to access care or to be open about these aspects of their identity during a visit. Participants offered suggestions acknowledging that if providers were more personable, they might be more willing to disclose.

“*I feel like doctors could be just a little bit more personable with their clients…*.*I’m not saying that we have to be best friends*. *Although that would be lovely too*. *But I feel like if I’m telling you all my deepest darkest whatever*, *and I’m letting you prick and poke and touch my body and all these things*, *there has to be some level of… I don’t want to go to the doctor and feel like I’m going to see a client*. *I don’t know*, *I just feel like maybe doctors could be just a little bit more personable*.”

(IDI 20, Multiracial, Transwoman, 34 year old)

#### Financial barriers to accessing services

Participants shared that financial barriers to comprehensive healthcare were often unsurmountable. Even participants with health insurance reported that consistent psychotherapy, regular medications and dental care were cost prohibitive. Some participants spoke of sliding-scale programs yet found that they were still cost prohibitive. As one participant stated:

“*Even like when sliding scale became more common*, *even that I was like*, *I can’t reach your minimum*, *and of course if you’re – I think when you’re approached with like preventative care versus food*, *you have to choose food*.”

(IIDI 08, white gender queer, 28 year old)

Due to the criminalization of sex work, many participants in the study also reported not having access to health insurance or government assistance. Access to mental health services and preventative healthcare is cost prohibitive without insurance. One participant acknowledged how psychotherapy had been helpful in the past, but how she no longer has the resources to receive counseling:

“*I don’t have health insurance*. *And I find it to be too expensive […] if healthcare was more affordable*, *but as an independent sex worker/a contractor…you know*, *I don’t have that[…]I see a counselor on an occasional basis*. *It’s something I used to do more regularly*, *but it just got too expensive*.”

(IDI 12, white ciswoman, 30 year old,)

Another participant expressed this same financial burden that was further compounded by intersectional identities/needs, such as race and socioeconomic status (SES):

“*Honestly money and being able to access a therapist on a regular basis*, *not having insurance*, *finding a Black therapist*. *These are the things that*, *for me are the obstacles*.”

(IDI 19, Black gender fluid, 32 year old)

Most participants described lacking finances as a critical barrier to accessing care related to their sexual health needs.

#### Openness & shared understanding

The participants who reported positive experiences with healthcare providers acknowledged feeling seen and understood by their provider. Rather than experiencing work-related stigma or judgment about sexual practices experienced by the majority of participants, patients who were met with openness and a sense of shared understanding reported trusting and liking their care provider because questions felt judgment-free and consistent with their health needs. Participants also discussed how care provider affirmation of their multiple intersecting identities supports a feeling of safety within the healthcare setting.

“*They ask me questions and make sure I’ve got what I need*. *Every time I go in […] it’s always like*, *hey*, *you know*, *how is work going*, *do you feel safe at work*, *you know*, *have you had any clients that you’re worried about*? *Like they’re always super supportive*.”

(IDI 4, white ciswoman, 39 year old)

Participants who reported that their having multiple sex partners were met with respect by their care providers described care providers responses as comfortable, appropriate and patient-centered. One participant described how her provider seemed accepting in her response to her disclosing her sex work.

“*So my most recent therapist that I had was the first therapist that I ever had that I was completely forward and honest with about being sex worker*, *and I was really lucky that she responded positively to it*. *And I didn’t feel judged*, *and I felt like it was safe space and you know she wasn’t necessarily a provider who had experienced working with sex workers*, *but just the fact*, *knowing that it was a safe space that I wasn’t being judged that made all the difference for me*.”

(IDI 2, Mixed Race, Ciswoman, 26 year old)

Another participant expressed a noted change over time, acknowledging that some providers have become more accepting of patients who disclose their sex work:

“*Last time I saw her*, *you know*, *she’s like*, *so how are you doing with using or*, *you know*, *are you working*, *you know*, *trying to like figure out like where I’m at*, *you know*, *and would never*, *I feel like she would never like chastise me or judge me or criticize me or anything that you know*, *whereas*, *years and years ago*, *like*, *oh my god*. *like hide it*. *You know*, *like it’s the plague*.”

(IDI 05, Latinx ciswoman, 25 year old)

#### Acceptance of intersectional identities

Participants noted the intersectionality of identities (i.e., race, gender and sexual orientation) and how providers treat them based on these identities. One participant discussed that they were more likely to share accurate medical histories with providers who provided patient-centered care by exhibiting cultural competencies regarding other types of marginalized identities and their intersections with sex work.

“*You know*, *[the provider] being LGBTQ friendly and kind of almost automatically – that kind of makes them pretty sex worker friendly for the most part*”

(IDI 06, white transwoman, 30 year old)

Participants who felt accepted and understood by their providers were willing to engage in regular healthcare. Participants discussed how they felt respected and empowered within community when shared identities (e.g., race, sexual orientation) aligned with clinic staff. One participant directly acknowledged the need for healthcare provider training around intersectional identities and the lived experiences of people who engage in sex work:

“*There needs to be more training around and mandatory mandated trainings around cultural understanding*, *queer understanding*, *there needs to be an intersectional mandate[…] understanding that different people need different things*. *Some stuff is just cultural […] that is just an acknowledgment in a way that people survive*.”

(IDI 19, Black, gender fluid, 32 years old)

Overall, participants who reported positive healthcare experiences described providers who create safe spaces for clients by listening, by asking about pronouns, by wearing or hanging visual representations of inclusivity and by conveying interest in patients as individuals. In essence, care that aligns with the tenets of cultural safety was seen as beneficial by participants. When talking about care received at Howard Brown Health, an LGBTQ+ affirming Federally Qualified Health Center, one participant described culturally safe care by stating:

“*Is it like from the first time I interacted with them (provider) to every single interaction every single visit I’ve had with them is just…*. *their bedside manner and you know whatever training they go through to be I don’t know*, *just like culturally aware of queer people and sex workers and all these things*. *It’s just a quality of care that I think as a queer person*, *as a sex worker is really rare to find*.

(IDI 12, white, ciswoman, 30 year old)

#### Alternatives to formal healthcare

In response to lacking acceptance of intersectional identities, participants reported bypassing formal healthcare systems and navigating alternative ways to practice wellness that were more accepting, accessible, and felt emotionally safe. Participants discussed daily rituals which included yoga, exercise, strength training and meditation. These regular practices along with healthful eating and minimizing use of substances were viewed not only as a way to stay healthy but also as a strategy to avoid seeing a care provider. One participant described seeking care or going to see a healthcare provider as a last result:

*I do feel like I just assume that diet is medicine*, *which a lot of people say*, *and that’s hard to figure out too…But if I am sick*, *it’s the last resort to think of going to the doctor*. *And sometimes*, *it’s not even an option*, *but even when it is*, *I’m just thinking about what foods you eat*, *I don’t know*. *Looking up anything holistic online*, *anything…I feel like the most solid form of self-protection for your health that I can think of is to exercise and try to eat well*. *So I’m like*, *okay*, *if I can just do that then*, *yeah*, *I’m safe*.

(IDI 17, white, genderqueer, 31 year old)

Alternatives to formal healthcare included several home wellness practices, such as yoga, meditation, exercise:

“*I carry a lot of stress on my back and my chest*. *And that’s where working out daily and meditation helps*. *I try to take care of things in a more natural way*. *I feel like that works more for me*.”

(IDI 5, multiracial ciswoman, 34 years old,)

“*Um*, *well*, *I really like working out*. *[…] I was doing yoga and longboarding and*, *you know*, *doing weight training*. *Definitely a lot more active and then just watching what I eat*, *or*, *you know*, *doing my best because I can be an emotional eater*, *but trying to get like all the nutrients right and not overeating*.”

(IDI 10, multiracial ciswoman, 27 year old)

#### The power of community

Consistent with community empowered interventions, finding a sense of community was also referenced as health-supportive. Participants acknowledged the need to connect with others who do the work they do, through sharing experiences of vetting, safety precautions, bad dates, coping mechanisms, and decompressing in community with one another. This participant relies on her chosen family for support:

“*When you’re when you’re just trying to exist as a woman*, *and you’re also being kind of fetishized by this component that is making it difficult to be seen as such definitely was emotionally taxing*. *But*, *I think I think my most helpful thing for maintaining health again is just having a queer and trans family for me*. *You know*, *people that I can be with and move away from that those specific spaces because those spaces are like*, *you know*, *again*, *it’s all mostly older cis*, *white men most of them and you know*, *that is a very weird space because it is very reflective of this kind of cis-het normative structure … But like*, *because there is a desire for normality*, *a little bit sometimes because of your that accented abnormality of who you are in your body*. *So creating a boundary and that space between my clients and that performance*. *Yeah*, *that’s been I think that’s like probably the main thing for my mental health*. *Having having a community in the secular community doesn’t*, *that’s the biggest one*. *I mean*, *yeah that’s*, *that’s*, *that’s massive to have other sex worker friends to talk to you about your job*. “

(ID6, white transwoman, 30 year old)

The promise of community is not only evident in research supporting the effectiveness of community empowered interventions in reducing HIV among sex workers, but this longing for supportive community was apparent across all epidemiological contexts within this study.

*I have a subset of friends that don’t know (about the sex work)*, *and those are mostly friends that are related to my day job[…*.*]I can’t be open with all of my friends*.

(IDI 14, white ciswoman, 38 year old)

Participants acknowledged how peer facilitated support groups and engaging with peers around sex work was empowering because it was in contrast to their regular experiences of hiding essential components of their lives. For example, one participant acknowledged how grateful she was that a trusted peer would host regular gatherings to support community empowerment.

I love how *** has brunches or used to have brunches every month. *That was great*, *because just meeting other providers and not feeling alone and just kind of seeing how we’re all kind of into this together and everybody’s super sweet*.

(IDI 15, Asian ciswoman, 34 year old)

They discussed appreciating online and in-person opportunities to connect and feel seen and understood on various formats.

## Discussion

Utilizing these qualitative findings to guide future interventions is a necessary step toward reducing barriers to care for sex workers in Chicago. Further, community empowered, culturally safe care aims to address the specific health, social, cultural needs of each patient so that all people can attain their ideal level of health. Our findings reverberate the call for culturally safe [18,22,23] and community empowered approach to care [3,12,26]. These findings also demonstrate how patient-centered care for sex workers might include independent holistic wellness exercises, accessible pay scales for services, community empowered HIV/STI prevention, and respectful and supportive physical and mental health promotion.

Our findings align with previous research on barriers to effective healthcare with sex workers, including how stigma operates in clinical settings to reduce patient-centered care among this population [3,33,34]. Effective patient-centered care is dependent on open communication, trust, and shared decision making between patient and provider [19,35,36]. Results from our study illustrate how stigma toward sex workers promotes a lack of trust in both patients and providers and inhibits effective communication and care. As other research has shown, when medical providers are uncertain or ambivalent within a medical encounter, it upsets the normal power dynamic in the patient-provider relationship and leads to interpersonal stigma, to reinforce the power and authority of the medical provider in these interactions [33]. Providers are encouraged to engage in a practice of purposeful self-reflection to become aware of implicit bias, uncertainties, and power-dynamics that may be at play in order to create a culturally safe patient encounter [18]. This self-reflection can and should include tools for providers that help to build an awareness about how multiple aspects of identity (race/ethnicity, gender, sexual orientation, type of employment) multiply and are intersectionally influenced by the systems of oppression (racism, heteropatriarchy, cisgenderism, and capitalism) that either provide privilege and opportunity for certain identities, while creating marginalization and constraining opportunity for other identities [37].

The emergence of alternative forms of care and self-care reported by our study participants merits further examination and further research. In the absence of affirming medical care, seeking out alternative models of care has been previously reported among populations experiencing social marginalization [34,38] and lack of support from their medical providers [39]. Sharing experiences of alternative and self-care among sex workers may inform the development of community and community-empowered interventions.

### Implications

Community Empowered Continuing Education for care providers is a logical step toward health equity for marginalized and vulnerable populations. Leadership, innovation, inclusion, and integrity are values which must guide patient-centered, culturally safe care [22]. The goals of cultural safety go beyond recognizing disparities, and instead challenge systems that create inequality by focusing on provider-patient power dynamics as a source of this inequality [18]. In order to incorporate the stated personalized health strategies sex workers are already using, patient-centered healthcare must be responsive and recognize that patients are the experts in their own lives [18]. Patient interactions with healthcare providers, as described by most sex worker participants, illustrate a lack of understanding about the lived experience of those engaged in sex work. For the few who reported positive interactions with care providers, it is important to highlight their experience to inform future provider trainings. A training, developed in collaboration with sex workers and guided by a cultural safety framework, would seek to improve clinician skills when caring for sex workers.

#### Community empowerment and sex work

Community empowerment models of care concretely address the structural and social barriers keeping sex workers, like those in this study, from needed information, services, and treatments. The criminalization of sex work and the resultant stigma attached has implications for how or even whether those engaged in sex work access healthcare. For us as healthcare providers, our own personal beliefs and values inform how we interact with our patients including those engaged in sex work. Reflexivity is key to establishing trusting relationships with our patients that ensures that those engaged in transactional sex are not hesitant to seek care on account of how they will be treated. Healthcare providers’ awareness about how power operates within the provider-patient encounter will disrupt the multiple intersecting oppressions that participants identified played a role in unsafe healthcare encounters. Healthcare providers can play an important advocacy role on behalf of their patients to ensure that those engaged in sex work receive culturally safe care that addresses their unique health needs.

### Limitations

Our findings are limited by the potential for bias present in a small qualitative study. Interviews were completed during Illinois’s strict first phase of shelter-in-place where schools and all non-essential businesses were closed due to COVID-19. This highlights the notion that those who were able to participate had access to a device and to the internet. As such, those who may not have access to a smart phone or reliable internet were unable to participate in this study thereby limiting the reach of the work. Recruitment for the study was done online and may have impacted the potential for bias toward those who engage with social media. The level of education further demonstrates that this sample may be missing those most vulnerable. A sample of predominantly white/cis women supports the need for an additional follow-up study focused on the specific needs of Black sex workers. Additionally, all interviews for this study were conducted in English. As such, we clearly did not access the experience of sex workers for whom English may not be the primary language.

## Conclusion

Responding to the stated needs of the patient population by developing community empowered care has the potential to promote health, prevent disease and improve quality of life for those most marginalized [18,26,40,41]. In so doing, a uniquely responsive, patient-centered model of care that takes into consideration the intersectional needs of sex workers may serve as a change agent toward social justice and health equity [12,41–43]. Additionally, continuing education around sex work for healthcare providers would help to mitigate the stigma and negative experiences with healthcare providers so frequently reported by participants. A training for care providers, designed in partnership with sex workers, offers an empowering step toward health equity for those engaged in sex work. In keeping with cultural safety and community empowerment, such a training requires the care provider to commit to learn about the heterogeneity within sex work, to self-reflect on biases and values and to determine how best to ask questions that are nonjudgmental or assumptive in relation to both personal and professional sexual behavior. The specific experiences of seeking care as a Black sex worker must further inform future trainings, which will require focused efforts and collaborations to engage and center the voices of Black sex workers. To that end, future studies informed by intersectionality as a framework could offer greater insight into the ways that multiple systems of oppression work to create a matrix of constrained opportunities that shape the unique realities and health needs of sex workers who occupy multiple social locations or identities on the basis of race, class, gender and sexual orientation [37,44]. Supporting care providers to gain an understanding of various needs and resources to promote the health of sex workers encourages respectful and individualized care of those most marginalized and vulnerable. The research team members are working with Howard Brown Health to develop and ultimately test such a training.

## Supporting information

S1 Appendix COREQ checklist. (DOCX)

S1 Table Frequency of salient healthcare experiences themes. (DOCX)
