# Supplementary material for: “Feeling Safe, Feeling Seen, Feeling Free”: Combating stigma and creating culturally safe care for sex workers in Chicago
Source: PLoS One. 2021 Jun 29;16(6):e0253749. doi: 10.1371/journal.pone.0253749 (PMC8241054; doi:10.1371/journal.pone.0253749)
Supplement: S1 Appendix — (DOCX) [file pone.0253749.s001.docx]

| **S1 Appendix. COREQ Checklist**  **No**  **Domain 1: Research team and reflexivity** | Item | Notes |  |
| --- | --- | --- | --- |
| Personal Characteristics |  |  |  |
| 1. | Interviewer/facilitator | The identities of the interviewers are protected because of their relationship to the sex work community. A subset of interviewers were shadowed by author Randi Singer to ensure fidelity to the semi-structured interview guide. |  |
| 2. | Credentials | PhD, MSN, CNM, MEd, RN |  |
| 3. | Occupation | Clinical Assistant Professor of Nursing, University of Illinois at Chicago |  |
| 4. | Gender | Female |  |
| 5. | Experience and training | Randi Singer, PhD, MSN, MEd, CNM, RN is an Assistant Clinical Professor at the University of Illinois Chicago College of Nursing. Dr. Singer obtained her BA from Clark University, her MSN from Vanderbilt University and her PhD from Widener University. A CNM with a background in human sexuality, social justice and equity education for healthcare providers, Singer’s research and practice have focused on how reproductive and sexual health education through an equity lens can reduce health disparity for vulnerable and marginalized populations such as LGBTQIA pregnant and non-pregnant patients, sex workers,1st generation LatinX adolescents and pregnant teens. |  |
| Relationship with participants |  |  |  |
| 6. | Relationship established | Participants were recruited through community organizations with appropriate rapport, including Sex Worker Outreach Project-Chicago (SWOP), Howard Brown Health (HBH), Centering Healthcare Institute (CHI) |  |
| 7. | Participant knowledge of the interviewer | Participants knew that their interviewers were either current or former sex workers, and that the supervising researcher had a history of working with the sex work community. |  |
| 8. | Interviewer characteristics | Researchers recruited peer interviewers to reduce anti-sex work bias and increase understanding of the sex work community, its jargon, and its shared experiences. |  |
| **Domain 2: study design** |  |  |  |
| Theoretical framework |  |  |  |
| 9. | Methodological orientation and Theory | Content analysis and grounded theory were used to inform the analysis. |  |
| Participant selection |  |  |  |
| 10. | Sampling | Participants were recruited using convenience and snowball sampling. Recruitment hubs included those listen in item 6 and recruitment efforts by peers in the sex work community who served as members of the research team. |  |
| 11. | Method of approach | Participants were recruited through clinic-based flyers, social media, listserves and other outreach activities. |  |
| 12. | Sample size | N=21 |  |
| 13. | Non-participation | N=0 |  |
| Setting |  |  |  |
| 14. | Setting of data collection | Participants were interviewed via Zoom. |  |
| 15. | Presence of non-participants | No |  |
| 16. | Description of sample | Of the sex workers interviewed, 52% (n = 11) identified as either Black, Latinx, American Indian or Asian American, with 48% (n = 10) identifying as white. A total of 52% (n = 11) identified as cisgender women, 33% (n = 7) identified as transgender or gender fluid, 10% (n = 2) identified as cisgender men, and 5% (n = 1) declined to answer. The majority (81%, n = 17) identified as either queer, bisexual, pansexual, or gay, 14% (n = 3) participants identified as heterosexual and 5% (n = 1) identified as asexual. |  |
| Data collection |  |  |  |
| 17. | Interview guide | The interview guide was informed by the literature and previous research, and covered physical, sexual and emotional health, experiences with healthcare, HIV/STI prevention, and harm reduction techniques. The semi-structured interview guide contained questions and example probes but allowed interviewers to add probes as needed and to explore topics naturally as they emerged. |  |
| 18. | Repeat interviews | No |  |
| 19. | Audio/visual recording | Interviews were audio recorded using Zoom for later transcription. |  |
| 20. | Field notes | Yes, field notes were recorded during interviews. Analyses in this paper are based only on participant data, not researcher field notes. |  |
| 21. | Duration | 45-90 minutes |  |
| 22. | Data saturation | Yes. A second round of recruitment is currently underway to ensure saturation of data regarding the Black sex work community, specifically. |  |
| 23. | Transcripts returned | No |  |
| **Domain 3: analysis and findings** |  |  |  |
| Data analysis |  |  |  |
| 24. | Number of data coders | 2 |  |
| 25. | Description of the coding tree | The codebook was provided for review to the research team and was drawn directly from the interview guide to support rapid content analyses. |  |
| 26. | Derivation of themes | Themes were derived from the data and reviewed for accuracy by the research team, which included current and former sex workers. |  |
| 27. | Software | Dedoose Qualitative Analysis Softare |  |
| 28. | Participant checking | No |  |
| Reporting |  |  |  |
| 29. | Quotations presented | Yes. Each quote is identified with an anonymous participant ID number to ensure participant safety. |  |
| 30. | Data and findings consistent | Data and findings are consistent and mutually reflective and have been assessed for validity. |  |
| 31. | Clarity of major themes | Yes |  |
| 32. | Clarity of minor themes | Minor themes were assessed by the research team for validity and relevance and are included here if and when relevant to the analysis. |  |

Adapted from the Consolidated criteria for reporting qualitative studies (COREQ): 32-item checklist.

Tong, A., Sainsbury, P., & Craig, J. (2007). Consolidated criteria for reporting qualitative research (COREQ): a 32-item checklist for interviews and focus groups. *International journal for quality in health care*, *19*(6), 349-357.
