# Supplementary material for: “Feeling Safe, Feeling Seen, Feeling Free”: Combating stigma and creating culturally safe care for sex workers in Chicago
Source: PLoS One. 2021 Jun 29;16(6):e0253749. doi: 10.1371/journal.pone.0253749 (PMC8241054; doi:10.1371/journal.pone.0253749)
Supplement: S1 Table — (DOCX) [file pone.0253749.s002.docx]

| Table 2. Frequency of Salient Healthcare Experience Themes | | |
| --- | --- | --- |
| Theme | Interview Count | Illustrative Quote |
| **Healthcare Stigma** | 16 | *Oh God, they can check their judgment at the goddamn door. Like that, that is, that's all they can do at this point, like, just stop it, turn it off. I'm going to tell you what I do and you're not gonna like it. And I'm still going to tell you.  I'm gonna have to tell the details and you're really not gonna like it. So maybe you should be in a different profession*. |
| **Disclosure** | 10 | *There are some people, a lot of people that are a lot more active sexually than I am, as a worker, so I don't feel like it should matter either way. I don't disclose that …* |
| **Dismissal, Disregard, Distrust** | 14 | *I think the most important thing is being listened to because then even if you, if you come in with a problem or complaint or something, even if they can’t fix it, they at least can validate you and come up with a plan or something to try and figure it out. Instead of, I think, yeah. Like dismissiveness is the biggest thing. And I don’t, I know they’re so busy and like it’s probably, it’s not personal probably, but I think a lot of doctors forget what it feels like to be on the other side.* |
| **Financial Barriers** | 18 | *It's so hard for people just to like get to the doctor without being a shitload of money in debt.* |
| **Openness & Shared Understanding** | 12 | *I was a little bit nervous in the beginning, but they made me really comfortable and just kind of normal. Like normalized in the sense where it was like not there... He could sense I was nervous talking about it and he was just like, oh, don't worry, it's not a big deal. We see a variety of patients here and so I was like, Oh okay, cool. So it wasn't as bad, I was kind of nervous, but then it wasn't as bad.* |
| **Acceptance of Intersectional Identities** | 6 | *Just like culturally aware of queer people and sex workers and all these things. It's just a quality of care that I think as a queer person as a sex worker is really rare to find like in most other places, you know, usually you only find that kind of like consciousness like actually asking people about their pronouns and not assuming, like hetero sexuality of a patient and stuff like that and just things like that. It's like also like, you know, as a queer person when I'm interacting with and like being served as like a patient like being served or being like taken care of by queer people and people obviously within the queer community and identify within the community and having name tags with their pronouns and all that stuff on it is just like huge.* |
| **Alternatives to Formal Healthcare** | 15 | *I write and I dance so art has always been a solace for me. I believe in art therapy. I believe in using traumatic experiences and things that you’re experiencing that you may not have had that level of comfort to express it to someone else to pour into something that you may be passionate in. Whether it’s drawing or creative writing, dancing.* |
| **Community Support** | 14 | *I have like a community of people who want to talk about what your healthy sexual life looks like and I feel like if I just had if I just had a piece of that, I don't know if it would feel as hard as it does.* |
